# Tuning of Optical Stopband Wavelength and Effective Bandwidth of Gel-Immobilized Colloidal Photonic Crystal Films

**DOI:** 10.3390/gels9010056

**Published:** 2023-01-11

**Authors:** Ami Amano, Toshimitsu Kanai

**Affiliations:** Graduate School of Engineering Science, Yokohama National University, 79-5 Tokiwadai, Hodogaya, Yokohama 240-8501, Kanagawa, Japan

**Keywords:** colloidal crystal, photonic crystal, color tuning, Bragg reflection, hydrogel, photopolymerization

## Abstract

We show that both the optical stopband wavelength and effective bandwidth of films of gel-immobilized loosely packed colloidal photonic crystals can be controlled over a wide range. When the gelation reagent of the charge-stabilized colloidal crystals was photopolymerized under ultraviolet light using different upper- and bottom-light intensities, it resulted in a gel-immobilized colloidal crystal film with a broadened Bragg reflection peak. Moreover, the width of the Bragg peak increased from 30 to 190 nm as the difference between the light intensities increased. Films with wider Bragg peaks exhibited a brighter reflection color because of the superposition of the shifted Bragg reflections. Furthermore, the Bragg wavelength could be varied over a wide range (500–650 nm) while maintaining the same broadened effective bandwidth by varying the swelling solvent concentration. These findings will expand the applicability of colloidal crystals for use in photonic devices and color pigments.

## 1. Introduction

Colloidal crystals, which are monodispersed particles arranged periodically in three dimensions, show immense promise for use as photonic crystals [1,2,3,4,5,6,7]. Compared with photonic crystals fabricated by top-down processes, such as lithography and selective etching [8,9], colloidal photonic crystals have a significant advantage in that their optical stopband can readily be set in the visible-light region through a self-assembly process using submicron-sized colloids. Therefore, they are expected to find extensive use in various photonic applications and devices such as optical filters, lasers [10,11], sensors [12,13,14,15,16,17,18,19], color pigments [20,21], and displays [22,23]. For these applications, however, the control of the optical stopband, which is characterized by the stopband wavelength and bandwidth, is essential [24]. The wavelength and bandwidth of the stopband are experimentally observable as the wavelength and width of the Bragg reflection peak, respectively, in the reflection spectrum. The wavelength can be determined using Bragg’s law [24]:

(1) $$\lambda_{hkl}=2n_{c}d_{hkl}sin\theta$$

where *λ_hkl_* and *d_hkl_* are the Bragg wavelength and lattice spacing of the (*hkl*) plane, respectively; *n*_c_ is the refractive index of the colloidal crystals; and *θ* is the glancing angle. The Bragg wavelength can be varied over a wide range by changing the diameter and volume fraction of the particles, both of which have a determining effect on the lattice spacing. In particular, colloidal crystals that have a low packing density and are immobilized in a stimuli-sensitive hydrogel allow for the on-demand tuning of their lattice spacing and Bragg wavelength over a wide range via changes in the volume of the hydrogel by applying an external stimulus, such as a change in the temperature, pH, solvent, and mechanical stress [25,26,27,28]. However, although the Bragg wavelength is highly tunable, controlling the peak width over a wide range is difficult because the bandwidth at a particular particle volume fraction is intrinsically determined by the difference in the refractive indices of the particles and the matrix, which cannot be changed significantly.

An alternative approach to tuning the bandwidth is fabricating a graded structure. The changes in the lattice spacing and refractive index owing to variations in the dimensions effectively result in a wider stopband because of the superposition of the shifted stopbands [29,30]. We had previously reported that the effective stopband width of loosely packed colloidal crystals immobilized in a hydrogel film could be broadened by adjusting the photoirradiation conditions for photoinduced polymerization [31]. Charge-stabilized colloidal crystals, wherein charged particles form a crystalline structure with a low packing density in water owing to interparticle electrorepulsion, were immobilized in a hydrogel network by the photopolymerization of the gelation reagent dissolved in water [32,33,34,35,36,37]. For insufficiently low photoirradiation times, the effective stopband gradually expanded in the dark after irradiation. Furthermore, we observed that the stopband did not broaden further with additional irradiation [31]. Although this type of width broadening is essentially different from the expansion of the photonic bandgap in a perfect crystal, it is beneficial for practical applications because it has an equivalent effect on the apparent spectral properties.

In this study, we report a simple and practical method for tuning the effective bandwidth of gel-immobilized loosely packed colloidal photonic crystal films over a wide range. We demonstrate that when the gelation reagent of the charge-stabilized colloidal crystals is photopolymerized under ultraviolet (UV) light using different upper- and bottom-light intensities, it results in gel-immobilized colloidal crystals with a broadened Bragg reflection peak. Furthermore, we show that the Bragg wavelength can be altered over a wide range while keeping the broadened effective bandwidth unchanged by varying the concentration of the swelling solvent.

## 2. Results and Discussion

**Figure 1:**
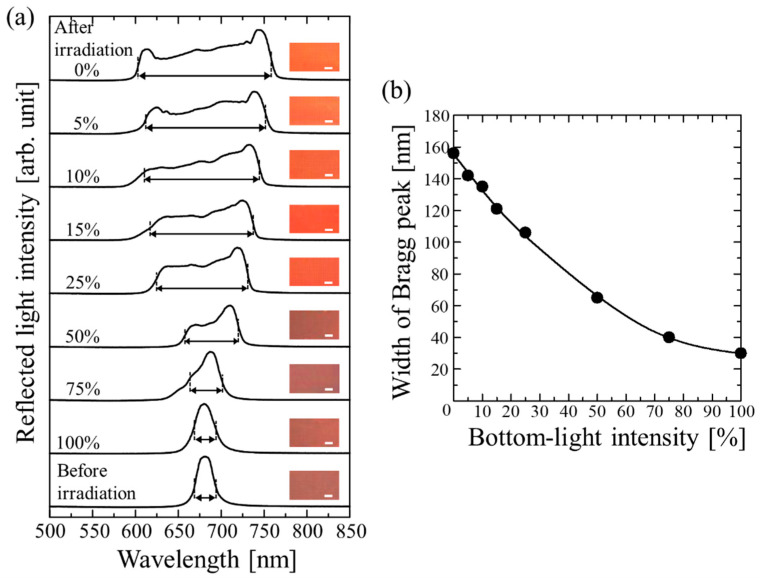
(**a**) Reflection spectra and photographs of colloidal crystals at normal incidence before and after UV light irradiation at different bottom-light intensities. Scale bar is 1.0 mm. (**b**) Width of Bragg peak of gel-immobilized colloidal crystal film as a function of bottom-light intensity. Width was defined as the difference between wavelengths corresponding to half intensities of short- and long-wavelength sides of the peak.

Figure 1a shows the reflection spectra and photographs of the colloidal crystals at normal incidence taken from the top of the cell before and after UV light irradiation at different bottom-light intensities. Before irradiation, a single reflection peak with a half-width of 30 nm is observed at 685 nm. Considering the Bragg condition, this peak was identified as the Bragg reflection from the (111) lattice plane of the face-centered cubic (fcc) structure, which was aligned parallel to the cell surface [38]. After UV light irradiation at upper- and lower-light intensities of 100%, no significant changes were observed in the position and intensity of the reflection peak. In other words, the colloidal crystals remained immobilized in the gel film and maintained their arrangement. In contrast, as the bottom-light intensity was reduced while keeping the upper-light intensity constant at 100%, the Bragg peak gradually broadened on both sides. Moreover, its color changed from dark red to bright orange. However, its large-area uniformity remained unaffected. At a bottom-light intensity of 0%, the width increased to 155 nm, which was 5.2 times that before irradiation (Figure 1b). A Bragg reflection this wide cannot be achieved using a straightforward approach for increasing the difference in the refractive indices of the colloidal particles and their surroundings.

**Figure 2:**
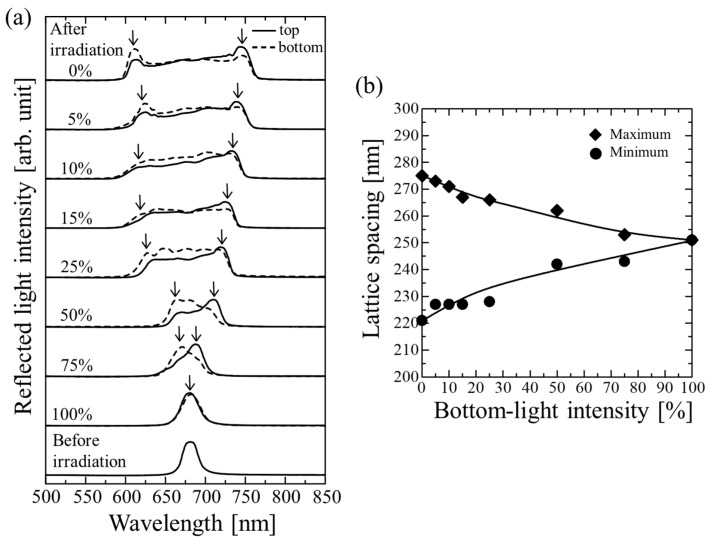
(**a**) Reflection spectra of gel-immobilized colloidal crystal films prepared using different bottom-light intensities as measured from top and bottom. Arrows indicate the positions of short- and long-wavelength sides of peaks. (**b**) Estimated maximum and minimum lattice spacings in the thickness direction as functions of bottom-light intensity.

Figure 2a shows a comparison of the reflection spectra measured from the top and bottom surfaces of the gel-immobilized colloidal crystal films prepared using different bottom-light intensities. At the bottom-light intensity of 100%, there were no significant differences in the spectra measured at the top and bottom. In contrast, at lower bottom-light intensities, the peaks in the spectra measured at the top exhibited higher- and lower-reflected light intensities on the long- and short-wavelength sides, respectively. This suggests that the lattice spacing of the fcc (111) plane was wider and narrower in the upper and lower parts of the films, respectively. The lattice spacing, *d*_111_, can be estimated by substituting the measured Bragg wavelength, *λ*_111_, at normal incidence (*θ* = 90°) in Equation (1):

(2) $$\lambda_{111}=2n_{c}d_{111}$$

The value of *n*_c_ can be approximated as

(3) $$n_{c}=n_{p}\phi_{p}+n_{\mathrm{pol}}\phi_{\mathrm{pol}}+n_{w}\phi_{w}$$

where *n*_p_, *n*_pol_, and *n*_w_ are the refractive indices of the polystyrene particles used (*n*_p_ = 1.59), the polymer in question (*n*_pol_ = 1.41), and water (*n*_w_ = 1.33), respectively. Furthermore, *ϕ*_p_, *ϕ*_pol_, and *ϕ*_w_ are the volume fractions of the polystyrene particles, polymer, and water, respectively, and exhibit the relationships *ϕ*_w_ = 1 − *ϕ*_p_ − *ϕ*_pol_ and *ϕ*_pol_ = 0.72 *ϕ*_p_. The latter was determined from the amount of the gelation reagent added to the colloidal suspension [14,18]. In addition, *ϕ*_p_ was determined based on the geometrical properties of the fcc structure using *d*_111_ and the particle diameter, *d*:

(4) $$\phi_{p}=\frac{2\pi }{9\sqrt{3}}\;{\left(\frac{d}{d_{111}}\right)}^{3}$$

Because the spectra of the gel-immobilized colloidal crystal film prepared using top- and bottom-light intensities of 100% were almost the same as those before polymerization, the lattice spacing, *d*_111_, was assumed to be constant throughout the film. By substituting the observed Bragg wavelength (685 nm) in Equation (2) and then using Equations (2)–(4), *d*_111_ was calculated to be 251 nm. For the gel-immobilized colloidal crystal film with a broadened Bragg peak, the maximum and minimum values of *d*_111_ were estimated by substituting the wavelengths corresponding to the long- and short-wavelength sides of the peak, respectively, in Equation (2) and then using Equations (2)–(4). The results are plotted in Figure 2b. As the bottom-light intensity was decreased, the difference between the maximum and minimum lattice spacings increased gradually. At the bottom-light intensity of 0%, the maximum and minimum lattice spacings were 275 and 221 nm, respectively. These values correspond to a variation of 21.5% ($\frac{275\;\mathrm{nm}-221\;\mathrm{nm}}{251\;\mathrm{nm}}\times 100$) in the thickness direction.

**Figure 3:**
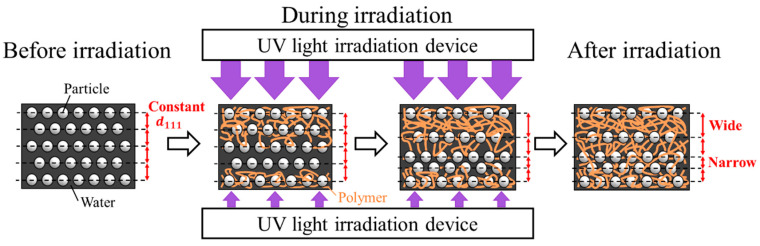
Schematic diagram of mechanism likely responsible for changes in lattice spacing in thickness direction during photopolymerization at different top- and bottom-light intensities.

We propose the following mechanism to explain the broadening of the Bragg peak. Before the UV light irradiation process, the polystyrene particles are arranged regularly in the water because of the electrorepulsive interaction between them. Hence, the lattice spacing perpendicular to the cell surface, *d*_111_, is constant throughout the suspension (Figure 3). When the intensities of both the upper and bottom lights are increased to 100%, the gel reagent dissolved in the water rapidly polymerizes both from the top and bottom at the same rate. Thus, the particle arrays are immobilized in the developed hydrogel network, resulting in a constant lattice spacing throughout the cell. However, when the bottom-light intensity is lower than that of the top light, polymerization occurs rapidly at the upper part. In this case, the polymer network is formed in the upper part and absorbs water from the undeveloped polymer network in the bottom part. This increases and decreases the lattice spacing in the top and bottom parts, respectively. As a result, the Bragg reflection is widened on both longer- and shorter-wavelength sides. Eventually, the particle arrangement is immobilized in the polymer network generated by photopolymerization. For providing the theoretical quantitative explanation, the examination of the degree of the non-homogeneous crosslinking via Fourier transform infrared spectroscopy may be beneficial [39,40].

**Figure 4:**
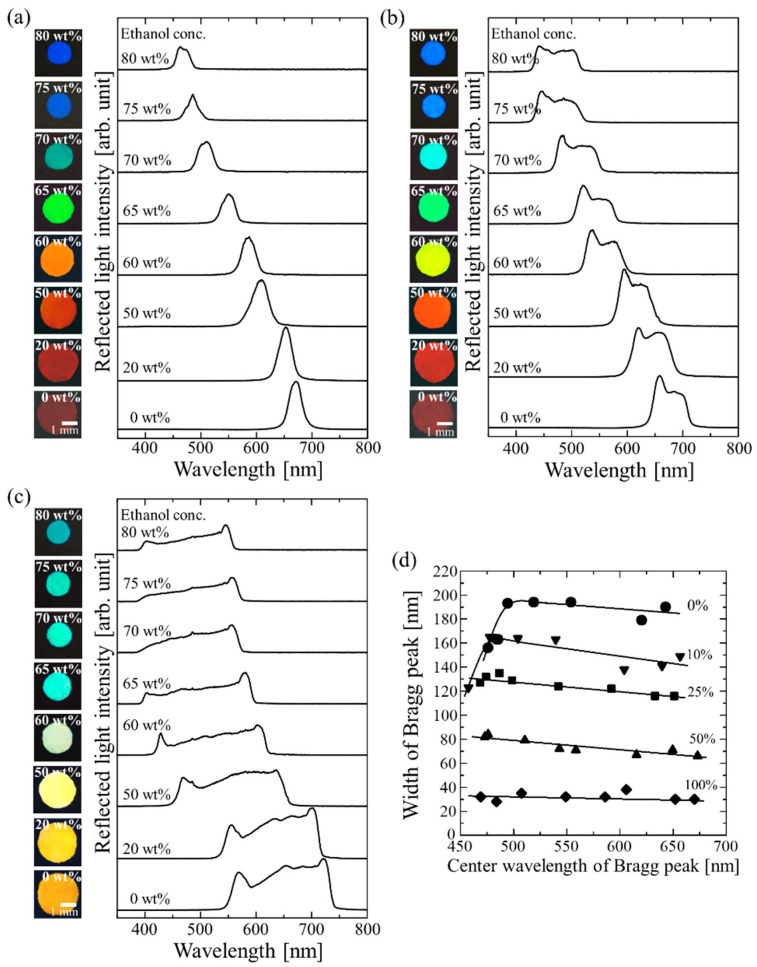
Photographs and reflection spectra of gel-immobilized colloidal crystal films prepared at bottom-light intensities of (**a**) 100%, (**b**) 50%, and (**c**) 0% in ethanol/water solutions with different ethanol concentrations. (**d**) Relationship between the width and center wavelength of Bragg reflection peak of gel-immobilized colloidal crystal films prepared at different bottom-light intensities.

In addition to the control of the effective width of the Bragg reflection peak, we tuned the Bragg wavelength by changing the concentration of the swelling solvent (ethanol). Figure 4a shows photographs and the reflection spectra of the gel-immobilized colloidal crystal film prepared at a bottom-light intensity of 100%. When the film was removed from the cell and placed in water, its Bragg wavelength blue-shifted by 15 nm while its peak width remained unchanged. Initially, the film gradually shrank with increasing ethanol concentration to 50 wt% and then shrank significantly, resulting in the following color changes: dark red (0 wt%), orange (60 wt%), light green (65 wt%), green (70 wt%), and blue (75 wt%). Thus, as the ethanol concentration was increased, the Bragg peak shifted to a shorter wavelength, which corresponded to a size reduction, while the peak width remained the same. The intensity of the reflected light gradually decreased. This was probably owing to the disordering of the particle arrangement because of the shrinkage of the surrounding gel. The film prepared at a bottom-light intensity of 50% exhibited a similar blue shift, as can be seen in Figure 4b. When the film prepared at a bottom-light intensity of 0% was soaked in water, the Bragg wavelength blue-shifted by 38 nm, and the width broadened from 155 nm to 190 nm; this width was 6.3-times that of the film prepared at the bottom-light intensity of 100%. The broadened Bragg peak underwent a blue shift with the increase in the ethanol concentration (Figure 4c). The films with Bragg peaks of different widths exhibited different colors and brightness values because of the superposition of the shifted Bragg reflection colors. Figure 4d shows the relationship between the width and center wavelength of the Bragg reflection peak as determined from the reflection spectra. The width and center wavelength lay in the 30–190 and 500–650 nm ranges, respectively. A reduction in the peak width of the gel-immobilized colloidal crystal films prepared at the bottom-light intensities of 0% and 10% was observed at wavelengths lower than the center wavelengths of 494 and 478 nm, respectively. This indicated that the reduction in the lattice spacing had reached its limit because the particles approached each other.

## 3. Conclusions

In summary, we demonstrated that both the optical stopband wavelength and effective bandwidth of films of gel-immobilized loosely packed colloidal photonic crystals can be tuned over a wide range. The difference between the upper- and lower-light intensities during photopolymerization induced variations in the thickness-direction lattice spacing of the charge-stabilized colloidal crystals immobilized within the gel film, resulting in an effective Bragg peak broadening from 30 to 190 nm. The maximum width would change depending on the film thickness. In addition, the Bragg wavelength could be altered over a wide range (500–650 nm) by varying the concentration of the swelling solvent; the effective bandwidth remained wide during this process. Colloidal crystals with a broadened Bragg peak exhibit different colors and brightnesses because of the superposition of the shifted Bragg reflection colors. These findings will significantly expand the scope of colloidal crystals for use in photonic devices and color pigments.

## 4. Materials and Methods

An aqueous suspension of monodispersed polystyrene particles with a diameter of 160 nm (Thermo Fisher Scientific, 5016 B, Waltham, MA, USA) was deionized using a mixed-bed ion-exchange resin (Bio-Rad, AG501-X8(D), Hercules, CA, USA). The resulting charge-stabilized colloidal crystals were centrifuged, and the supernatant was removed to increase the particle concentration. The gelation reagent was prepared by dissolving *N*-methylolacrylamide (NMAM) (FUJIFILM Wako Pure Chemical Corp., Tokyo, Japan) (used as the monomer), *N*,*N*′-methylenebisacrylamide (BIS) (FUJIFILM Wako Pure Chemical Corp., Tokyo, Japan) (used as the cross-linker), and 2,2′-azobis [2-methyl-*N*-(2-hydroxyethyl)propionamide] (VA) (FUJIFILM Wako Pure Chemical Corp., Tokyo, Japan) (used as the photoinitiator) in ultrapure water (Merck KGaA, Milli-Q system, Darmstadt, Germany). The gelation reagent and ultrapure water were then added to the condensed charge-stabilized colloidal crystals such that the NMAM, BIS, VA, and particle concentrations were 800 mM, 40 mM, 0.35 mM, and 10.4 vol%, respectively. The resulting solution was bubbled with Ar gas for 5 min and then shear-flowed into a flat capillary cell (channel height: 0.1 mm, width: 9 mm, length: 50 mm) to form a single-domain crystal. UV light from UV light irradiation devices (MORITEX SCHOTT, MBRL-CUV7530, Saitama, Japan) was uniformly irradiated from both the top and bottom sides of the cell through light diffusers for 90 min to polymerize the dissolved gelation reagent. The light intensity of the upper light device was set to the maximum in all the cases, whereas that of the bottom light device was reduced from 100% to 0% of the maximum. The reflection spectra of the colloidal crystals at normal incidence before and after UV light irradiation were measured both at the top and bottom using a fiber spectrometer (Soma Optics, Fastevert S-2630, Japan), and the crystals were photographed using a charge-coupled device (CCD) camera (Sony, XCD-V60CR, Tokyo, Japan).

Next, the thus-prepared gel-immobilized colloidal crystal film was removed from the cell and cut into discs 3 mm in diameter. These discs were immersed in ethanol/water solutions with different ethanol concentrations for approximately 24 h to replace the swelling solvent of the gel. The discs were then placed between two glass slides using Parafilm^TM^ (Bemis Flexible Packaging, Neenah, WI, USA) as the spacer, and their reflection spectra at normal incidence were measured using fiber spectrometers (Soma Optics, Fastevert S-2431 and S-2630, Tokyo, Japan). The discs were also photographed using a CCD camera (Sony, XCD-V60CR, Tokyo, Japan).

## Data Availability

The data presented in this study are available on request from the corresponding author.

## References

[1] Pieranski P. (1983). Colloidal Crystals. Contemp. Phys..

[2] Xia Y., Gates B., Yin Y., Lu Y. (2000). Monodispersed Colloidal Spheres: Old Materials with New Applications. Adv. Mater..

[3] Yoshino K., Kawagishi Y., Ozaki M., Kose A. (1999). Mechanical Tuning of the Optical Properties of Plastic Opal as a Photonic Crystal. Jpn. J. Appl. Phys..

[4] Baba T. (2008). Slow Light in Photonic Crystals. Nat. Photonics.

[5] Hou J., Li M., Song Y. (2018). Patterned Colloidal Photonic Crystals. Angew. Chem. Int. Ed..

[6] Clough J.M., Weder C., Schrettl S. (2021). Mechanochromism in Structurally Colored Polymeric Materials. Macromol. Rapid Commun..

[7] Lee G.H., Han S.H., Kim J.B., Kim D.J., Lee S., Hamonangan W.M., Lee J.M., Kim S. (2020). Elastic Photonic Microbeads as Building Blocks for Mechanochromic Materials. ACS Appl. Polym. Mater..

[8] Lin S.Y., Fleming J.G., Hetherington D.L., Smith B.K., Biswas R., Ho K.M., Sigalas M.M., Zubrzycki W., Kurtz S.R., Bur J. (1998). A Three-Dimensional Photonic Crystal Operating at Infrared Wavelengths. Nature.

[9] Noda S., Tomoda K., Yamamoto N., Chutinan A. (2000). Full Three-Dimensional Photonic Bandgap Crystals at Near-Infrared Wavelengths. Science.

[10] Furumi S., Fudouzi H., Miyazaki H.T., Sakka Y. (2007). Flexible Polymer Colloidal-Crystal Lasers with a Light-Emitting Planar Defect. Adv. Mater..

[11] Kim S.H., Kim S.H., Jeong W.C., Yang S.M. (2009). Low-Threshold Lasing in 3D Dye-Doped Photonic Crystals Derived from Colloidal Self-Assemblies. Chem. Mater..

[12] Weissman J.M., Sunkara H.B., Tse A.S., Asher S.A. (1996). Thermally Switchable Periodicities and Diffraction from Mesoscopically Ordered Materials. Science.

[13] Holtz J.H., Holtz J.S.W., Munro C.H., Asher S.A. (1998). Intelligent Polymerized Crystalline Colloidal Arrays: Novel Chemical Sensor Materials. Anal. Chem..

[14] Kanai T., Yano H., Kobayashi N., Sawada T. (2017). Enhancement of Thermosensitivity of Gel-Immobilized Tunable Colloidal Photonic Crystals with Anisotropic Contraction. ACS Macro Lett..

[15] Katsura C., Nobukawa S., Sugimoto H., Nakanishi E., Inomata K. (2017). Solvent-Responsive Coloring Behavior of Colloidal Crystal Films Consisting of Cross-Linked Polymer Nanoparticles. Colloid Polym. Sci..

[16] Foulger S.H., Jiang P., Lattam A.C., Smith D.W., Ballato J. (2001). Mechanochromic Response of Poly(ethylene glycol) Methacrylate Hydrogel Encapsulated Crystalline Colloidal Arrays. Langmuir.

[17] Fenzl C., Wilhelm S., Hirsch T., Wolfbeis O.S. (2013). Optical Sensing of the Ionic Strength Using Photonic Crystals in a Hydrogel Matrix. ACS. Appl. Mater. Interface.

[18] Tajima H., Amano A., Kanai T. (2021). Elastomer-Immobilized Tunable Colloidal Photonic Crystal Films with High Optical Qualities and High Maximum Strain. Mater. Adv..

[19] Miwa E., Watanabe K., Asai F., Seki T., Urayama K., Odent J., Raquez J.M., Takeoka Y. (2020). Composite Elastomer Exhibiting a Stress-Dependent Color Change and High Toughness Prepared by Self-Assembly of Silica Particles in a Polymer Network. ACS Appl. Polym. Mater..

[20] Kubo S., Gu Z.Z., Takahashi K., Fujishima A., Segawa H., Sato O. (2004). Tunable Photonic Band Gap Crystals Based on a Liquid Crystal-Infiltrated Inverse Opal Structure. J. Am. Chem. Soc..

[21] Wang H., Liu Y., Chen Z., Sun L., Zhao Y. (2020). Anisotropic Structural Color Particles from Colloidal Phase Separation. Sci. Adv..

[22] Arsenault A.C., Puzzo D.P., Manners I., Ozin G.A. (2007). Photonic-Crystal Full-Colour Displays. Nat. Photonics.

[23] Han M.G., Heo C.J., Shin C.G., Shim H.S., Kim J.W., Jin Y.W., Lee S.Y. (2013). Electrically Tunable Photonic Crystals from Long-Range Ordered Crystalline Arrays Composed of Copolymer Colloids. J. Mater. Chem. C.

[24] Holtz J.H., Asher S.A. (1997). Polymerized Colloidal Crystal Hydrogel Films as Intelligent Chemical Sensing Materials. Nature.

[25] Furumi S., Kanai T., Sawada T. (2011). Widely Tunable Lasing in a Colloidal Crystal Gel Film Permanently Stabilized by an Ionic Liquid. Adv. Mater..

[26] Iwayama Y., Yamanaka J., Takiguchi Y., Takasaka M., Ito K., Shinohara T., Sawada T., Yonese M. (2003). Optically Tunable Gelled Photonic Crystal Covering Almost the Entire Visible Light Wavelength Region. Langmuir.

[27] Kanai T., Sawada T., Yamanaka J., Kitamura K. (2005). Critical Concentration for Colloidal Crystallization Determined with Microliter Centrifuged Suspensions. Langmuir.

[28] Ryan C.C., Delezuk J.A.M., Pavinatto A., Oliveira  O.N., Fudouzi H., Pemble M.E., Bardosova M. (2016). Silica-Based Photonic Crystals Embedded in a Chitosan-TEOS Matrix: Preparation, Properties and Proposed Applications. J. Mater. Sci..

[29] Jiang P., Ostojic G.N., Narat R., Mittleman D.M., Colvin V.L. (2001). The Fabrication and Bandgap Engineering of Photonic Multilayers. Adv. Mater.

[30] Park J.H., Choi W.S., Koo H.Y., Hong J.C., Kim D.Y. (2006). Doped Colloidal Photonic Crystal Structure with Refractive Index Chirping to the [111] Crystallographic Axis. Langmuir.

[31] Kanai T., Sawada T., Toyotama A., Yamanaka J., Kitamura K. (2007). Tuning the Effective Width of the Optical Stop Band in Colloidal Photonic Crystals. Langmuir.

[32] Saito H., Takeoka Y., Watanabe M. (2003). Simple and Precision Design of Porous Gel as a Visible Indicator for Ionic Species and Concentration. Chem. Commun..

[33] Pagonis K., Bokias G. (2007). Temperature- and Solvent-Sensitive Hydrogels Based on N-isopropylacrylamide and N,N-dimethylacrylamide. Polym. Bull..

[34] Lee K., Asher S.A. (2000). Photonic Crystal Chemical Sensors: pH and Ionic Strength. J. Am. Chem. Soc..

[35] Sakiyama T., Takata H., Toga T., Nakanishi K. (2001). pH-Sensitive Shrinking of a Dextran Sulfate/Chitosan Complex Gel and Its Promotion Effect on the Release of Polymeric Substances. J. Appl. Polym. Sci..

[36] Toyotama A., Kanai T., Sawada T., Yamanaka J., Ito K., Kitamura K. (2005). Gelation of Colloidal Crystals without Degradation in Their Transmission Quality and Chemical Tuning. Langmuir.

[37] Zhang Y., Wang Y., Wang H., Yu Y., Zhong Q., Zhao Y. (2019). Super-Elastic Magnetic Structural Color Hydrogels. Small.

[38] Kanai T., Sawada T., Kitamura K. (2003). Optical Determination of the Lattice Constants of Colloidal Crystals without Use of the Refractive Index. Langmuir.

[39] Zhang X., Wang X., Li L., Wu R., Zhang S., Wu J., Wu W. (2015). A Novel Polyacrylamide-Based Superabsorbent with Temperature Switch for Steam Breakthrough Blockage. J. Appl. Polym. Sci..

[40] Călina I., Demeter M., Scărișoreanu A., Micutz M. (2021). Development of Novel Superabsorbent Hybrid Hydrogels by E-Beam Crosslinking. Gels.

